# PARP Inhibitor Upregulates PD-L1 Expression and Provides a New Combination Therapy in Pancreatic Cancer

**DOI:** 10.3389/fimmu.2021.762989

**Published:** 2021-12-17

**Authors:** Yali Wang, Kun Zheng, Hua Xiong, Yongbiao Huang, Xiuqiong Chen, Yilu Zhou, Wan Qin, Jinfang Su, Rui Chen, Hong Qiu, Xianglin Yuan, Yihua Wang, Yanmei Zou

**Affiliations:** ^1^ Department of Oncology, Tongji Hospital, Tongji Medical College, Huazhong University of Science and Technology, Wuhan, China; ^2^ Biological Sciences, Faculty of Environmental and Life Sciences, University of Southampton, Southampton, United Kingdom; ^3^ Institute for Life Sciences, University of Southampton, Southampton, United Kingdom

**Keywords:** pancreatic cancer, PARP inhibitors, pamiparib, PD-L1, CD8^+^ T cells

## Abstract

Despite recent improvements in treatment modalities, pancreatic cancer remains a highly lethal tumor with mortality rate increasing every year. Poly (ADP-ribose) polymerase (PARP) inhibitors are now used in pancreatic cancer as a breakthrough in targeted therapy. This study focused on whether PARP inhibitors (PARPis) can affect programmed death ligand-1 (PD-L1) expression in pancreatic cancer and whether immune checkpoint inhibitors of PD-L1/programmed death 1 (PD-1) can enhance the anti-tumor effects of PARPis. Here we found that PARPi, pamiparib, up-regulated PD-L1 expression on the surface of pancreatic cancer cells *in vitro* and *in vivo*. Mechanistically, pamiparib induced PD-L1 expression *via* JAK2/STAT3 pathway, at least partially, in pancreatic cancer. Importantly, pamiparib attenuated tumor growth; while co-administration of pamiparib with PD-L1 blockers significantly improved the therapeutic efficacy *in vivo* compared with monotherapy. Combination therapy resulted in an altered tumor immune microenvironment with a significant increase in windiness of CD8^+^ T cells, suggesting a potential role of CD8^+^ T cells in the combination therapy. Together, this study provides evidence for the clinical application of PARPis with anti-PD-L1/PD-1 drugs in the treatment of pancreatic cancer.

## Introduction

Pancreatic cancer is an extremely lethal disease with a poor prognosis. It ranks fourth and sixth, respectively, in causing cancer-related deaths in the USA and China (1), with a 5-year survival rate of less than 10% (2). Although surgery is the only treatment with curative potential, a few chemotherapeutic agents could improve the prognosis of the pancreatic cancer (3). For example, in addition to traditional chemotherapeutic agents such as gemcitabine or 5-fluorouracil, recent studies have shown that maintenance therapy with poly (ADP-ribose) polymerase (PARP) inhibitors is beneficial for patients with germline *BRCA* mutations and metastatic pancreatic cancer, and maybe a harbinger of progress in providing targeted therapy (4, 5).

PARP is a ribozyme involved in base excision repair, which transfers poly (ADP-ribose) (PAR) or mono-ADP-ribose to itself and/or other target proteins to sense and repair DNA damage (6, 7). Among the PARP protein family, PARP-1 has a primary role in the total activity and occupies a central position in the repair of DNA single-strand breaks (SSBs) (8, 9). PARP inhibitors (PARPis), whose most extensive mechanism of action is the inhibition on DNA damage repairing, have become promising for several cancer types, among which the clinical application of PARPis in ovarian cancer is the most advanced (10). Olaparib is the first PARPi approved by the Food and Drug Administration (FDA) for the treatment of advanced *BRCA*-dependent ovarian cancer (11). Pamiparib (BGB-290) is a highly selective PARP-1/2 inhibitor recently developed by BeiGene (Beijing) Co., Ltd (12). Its clinical trials in Chinese patients with advanced high-grade ovarian cancer and triple-negative breast cancer are in progress (13). In addition, PARPis have shown great potential in pancreatic cancer, and several clinical trials are underway to assess PARPis as monotherapy or combination therapy that would be clinically effective in the treatment of pancreatic cancer (14–17). Nevertheless, acquired resistance for PARPis has partially limited their use in clinical settings (18, 19). Breakthroughs in immune checkpoint blockade therapies represent an important turning point in cancer immunotherapy, deepening our understanding of tumor immune evasion (20). Programmed death 1 (PD-1) protein is a co-inhibitory receptor on the surface of activated T cells (21). One of its known ligands, programmed death ligand-1 (PD-L1), is selectively expressed on the surface of tumor cells and in the tumor microenvironment (22, 23). When PD-1 binds to PD-L1, activated T cells receive inhibitory signals and cease to produce anti-tumor immune responses (21), rendering PD-L1 a potentially promising target for the cancer immunotherapy (24, 25). However, immune checkpoint inhibitors are ineffective in pancreatic cancer, probably because PD-L1 expression is consistently low in various cell subsets of the pancreatic cancer (26–28).

In this study, we investigated the effects of PARPi, pamiparib, on pancreatic cancer and further explored its impact on the immune microenvironment. Here we found that pamiparib up-regulated PD-L1 expression on the surface of pancreatic cancer cells *in vitro* and *in vivo*. Mechanistically, pamiparib induced PD-L1 expression *via* JAK2/STAT3 pathway, at least partially, in pancreatic cancer. Importantly, pamiparib attenuated tumor growth; while co-administration of pamiparib with PD-L1 blockers significantly improved the therapeutic effect *in vivo* compared with monotherapy. Combination therapy resulted in an altered tumor immune microenvironment with a significant increase in windiness of CD8^+^ T cells, suggesting a potential role of CD8^+^ T cells in combination therapy. Together, this study provides evidence for the clinical application of PARPis with anti-PD-L1/PD-1 drugs in the treatment of pancreatic cancer.

## Results

### Pamiparib Affects Apoptosis, Cell Cycle, and Proliferation in Pancreatic Cancer Cells

**Figure 1:**
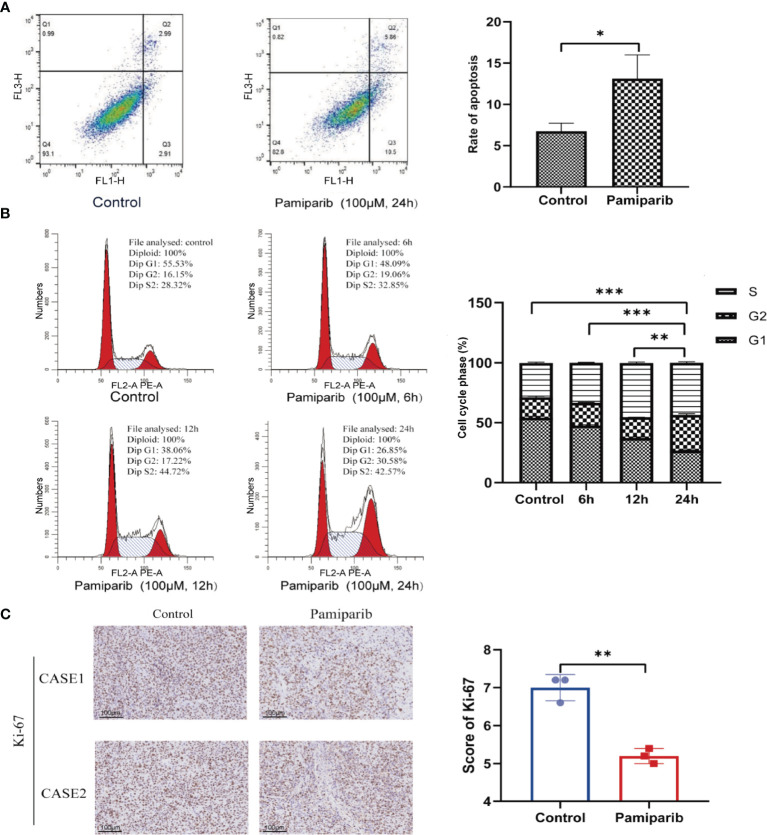
Pamiparib affects apoptosis, cell cycle, and proliferation in pancreatic cancer cells. **(A)** Flow cytometry detection of cell apoptosis showing pamiparib treatment causes a significant increase of apoptosis in SW1990 cells. **(B)** Flow cytometry detection of cell cycle showing pamiparib blocks SW1990 cells in G2/M phase. **(C)** IHC staining of Ki-67 showing pamiparib inhibits the proliferation of SW1990 cells in the *in vivo* environment. Data are mean ± SD; n = 3 samples per group. Scale bar, 100 μm. The IHC results were analyzed by Pearson χ2 test. *P < 0.05, **P < 0.01, ***P < 0.001.

To explore whether pamiparib could alter functionalities of pancreatic cancer cells, we treated SW1990 cells with pamiparib (100 μM). By flow cytometry assay, it was observed that the use of pamiparib significantly induced apoptosis of pancreatic cancer cells compared to the control group (*P* < 0.05; **Figure 1A**). Further detection of cell cycle distribution by flow cytometry revealed that SW1990 cells were significantly blocked in G2/M phase upon pamiparib treatment in a time-dependent manner (all *P* values less than 0.01; **Figure 1B**). This suggests that pamiparib can significantly induce apoptosis and block cell cycle progression of pancreatic cancer cells *in vitro*.

To check the *in vivo* effects of pamiparib, we inoculated SW1990 cells subcutaneously on the back of nude mice. Pamiparib was administered by gavage twice daily at a dose of 3 mg/kg for 2 weeks, followed by tumor tissues isolation and embedding. Immunohistochemistry (IHC) staining of Ki-67 in tumor sections demonstrated that pamiparib treatment significantly inhibited the proliferation of tumor cells *in vivo* (*P* < 0.01; **Figure 1C**).

### Effects of Pamiparib Treatment on PD-L1 Expression

**Figure 2:**
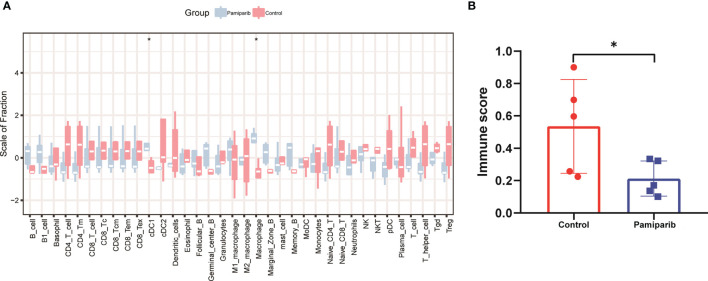
Bioinformatic analysis of RNA-seq dataset from a Pan-02 allograft tumor model treated with parmiparib. **(A)** Spectrograms of 22 immune cell expressions in 2 different groups. Control, control group; pamiparib, pamiparib monotherapy group. **(B)** Histogram of immune scores between the 2 groups. Control, control group; pamiparib, pamiparib monotherapy group. Data are mean ± SD; n = 5 samples per group. Data analysis was performed by unpaired *t*-test. *P < 0.05.

Acquired resistance for PARPis partially limits its use in the pancreatic cancer (18, 19). Their impacts on the immune microenvironment were investigated. We constructed a C57 mouse allograft tumor model using mouse pancreatic cancer cell line Pan-02, which was then gavaged with parmiparib. Tumor samples were excised for RNA sequencing (RNA-seq). CIBERSORT analysis was performed to calculate the abundance and immune fraction of 22 immune cells. A trend towards a suppressive effect on the expression of CD4^+^ T cells and CD8^+^ T cells was observed (**Figure 2A**). Using ESTIMATE calculations in R language, we found pamiparib treatment significantly reduced the immune score (*P* < 0.05; **Figure 2B**).

The results above suggested that pamiparib treatment could potentially lead to increased immunosuppression. Given the important role of PD-L1 upregulation in immunosuppression, we next investigated if pamiparib treatment in pancreatic cancer affects PD-L1 expression.

**Figure 3:**
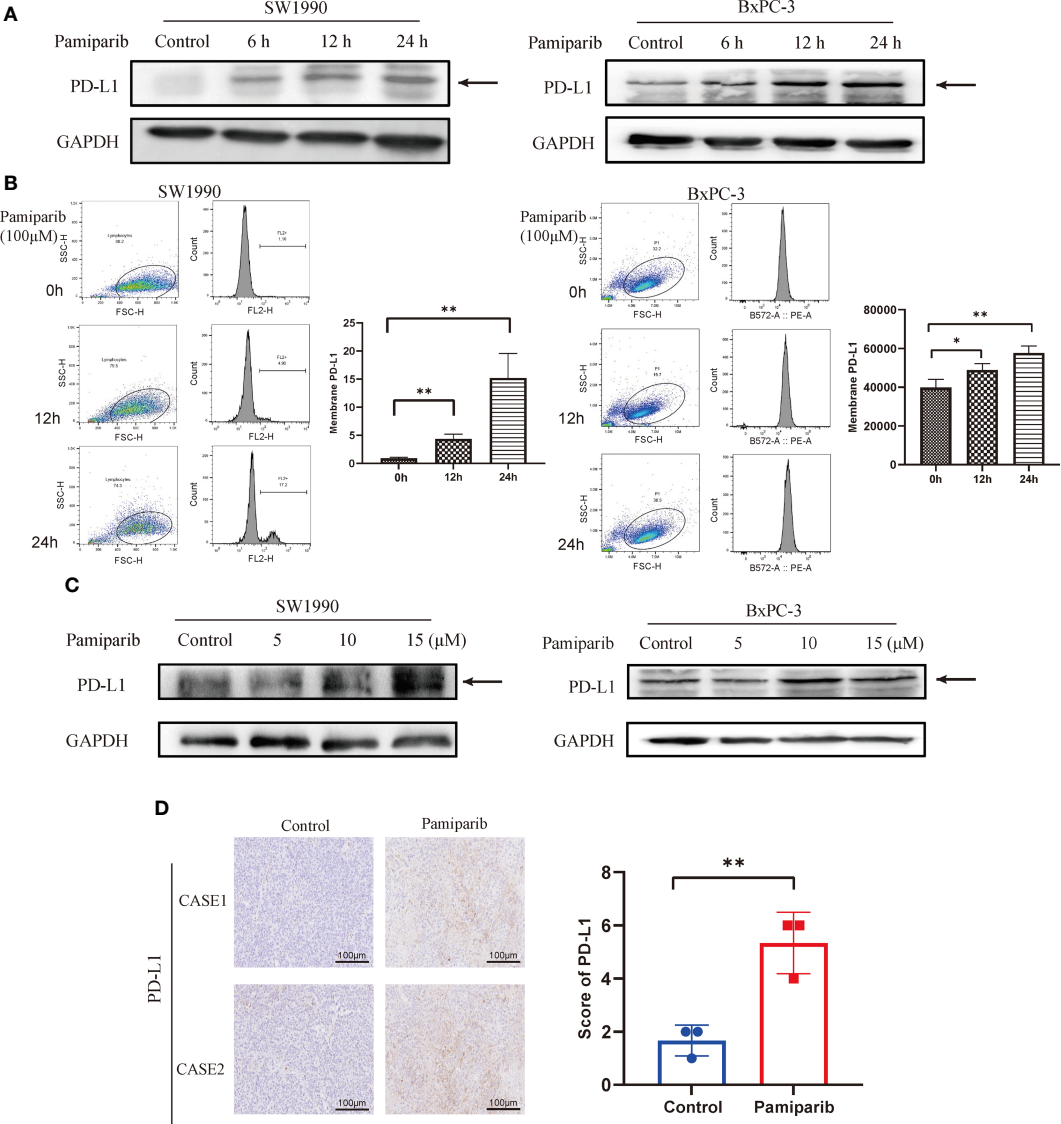
Effects of pamiparib treatment on PD-L1 expression. **(A)** SW1990 and BxPC-3 cells were treated with pamiparib, proteins were extracted at selected time points (0h, 6h, 12h, 24h), and PD-L1 protein expression was found to be up-regulated by immunoblotting. **(B)** Flow cytometry detection of PD-L1 expression on the surface of SW1990 and BxPC-3 cells after pamiparib treatment for different times. The flow cytometry results were analyzed by unpaired t-test. **(C)** PD-L1 protein expression was detected by immunoblotting after treatment of SW1990 and BxPC-3 cells with different concentrations of pamiparib for 24h (0, 5, 10, 15μM). **(D)** PD-L1 expression showed significant differences under IHC staining in control and nude mouse xenograft tumors treated with pamiparib. Control, control group; pamiparib, pamiparib monotherapy group. Data are mean ± SD; n = 3 samples per group. Scale bar, 100 μm. The IHC results were analyzed by Pearson χ2 test. *P < 0.05, **P < 0.01.

*In vitro*, we treated 2 different pancreatic cancer cells, SW1990 and BxPC-3, with pamiparib and PD-L1 expressions was examined by both immunoblotting and flow cytometry. After treatment of both cell lines with pamiparib (100 μM), the results showed that pamiparib significantly increased total PD-L1 protein levels in both cell lines in a time-dependent manner (**Figure 3A**). The results of flow cytometry showed that PD-L1 expression on the surface of pancreatic cancer cells increased with time of administration after treatment with pamiparib in both cell lines (all *P* values less than 0.05; **Figure 3B**). We also treated SW1990 and BxPC-3 cell lines with different concentrations of pamiparib for 24h and found that the treatment increased PD-L1 protein expression in pancreatic cancer cells in a dose-dependent manner (**Figure 3C**).

The efficacy of pamiparib treatment on PD-L1 expressions *in vivo* was further investigated in the SW1990 tumor model, in which SW1990 cells were inoculated subcutaneously on the back of nude mice. Pamiparib was administered to mice by gavage twice daily at a dose of 3 mg/kg for 2 weeks. Tumors were isolated from control or pamiparib-treated mice and stained for PD-L1 by IHC. The expression of PD-L1 was significantly higher in xenograft tumors of mice treated with pamiparib compared with untreated mice (*P* < 0.01; **Figure 3D**). Together, our results demonstrated that pamiparib treatment induces upregulation of PD-L1 expression in pancreatic cancer both *in vitro* and *in vivo*.

### Pamiparib Treatment Induces PD-L1 Expression *via* JAK2/STAT3 Pathway

To verify whether pamiparib-induced PD-L1 upregulation is required through the *PARP1* itself, we knocked down *PARP1* in SW1990 cells with siRNAs and treated the cells as followings: (i) control group; (ii) si*PARP1* group; (iii) pamiparib group; (iv) pamiparib + si*PARP1* group. Results suggested that PD-L1 expression was independent of *PARP1* levels (**Supplementary Figure 1A**). In order to find how pamiparib treatment regulates PD-L1 expression, we analyzed data from TCGA. We searched for pancreatic cancer in c-Bioportal, and genes that were positively correlated with PD-L1 (correlation coefficient ≥ 0.4) were identified and imported into STRING to construct a protein–protein interaction (PPI) co-expression network (**Supplementary Figure 1B**). KEGG enrichment (**Supplementary Table 1**) was performed to obtain pathways associated with PD-L1 expressions. We found that the enriched pathways included the NF-kB signaling pathway (false discovery rate, FDR = 0.0186), JAK-STAT signaling pathway (FDR = 0.00094), PI3K-AKT signaling pathway (FDR = 0.00014), and MAPK signaling pathway (FDR = 0.0014).

**Figure 4:**
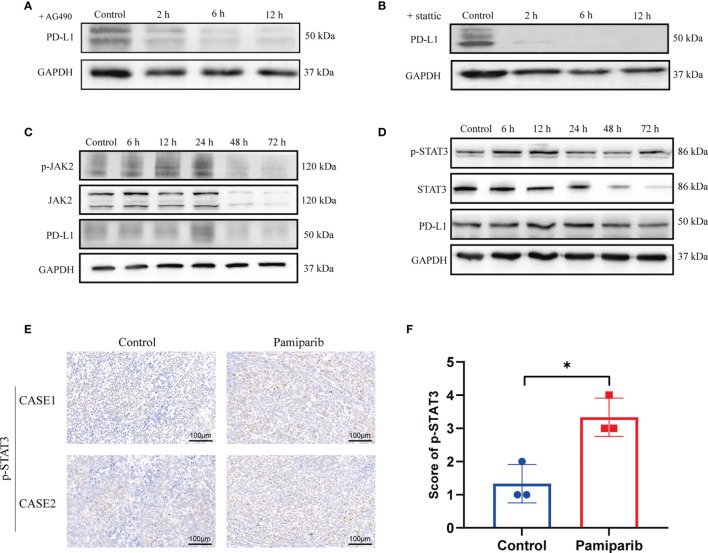
Pamiparib treatment induces PD-L1 expression *via* JAK2/STAT3 pathway. **(A)** Cells were pretreated with pamiparib (100 μM, 12h) and PD-L1 expression was assessed by immunoblotting after treatment with the concentrations (20 μM) of AG490 for 24h. **(B)** Cells were pretreated with pamiparib (100 μM, 12h) and PD-L1 expression was assessed by protein blotting after treatment with stattic (20 μM) for 24h. **(C)** Protein expression of phospho-JAK2 (p-JAK2), JAK2, and PD-L1 in SW1990 cells after being treated with pamiparib (100 μM) for the indicated times. **(D)** Protein expression of phospho-STAT3(p-STAT3), STAT3 and PD-L1 in SW1990 cells after being treated with pamiparib (100 μM) for the indicated times. GAPDH was used as a loading control. **(E)** IHC staining of p-STAT3 of nude mouse xenograft tumors in control group and treated with pamiparib group. Scale bar, 100 μm. **(F)** IHC staining score showed the expression of p-STAT3 was significantly upregulated in nude mice pamiparib treated group. Data are mean ± SD; n = 3 samples per group. The IHC results were analyzed by Pearson χ2 test. *P < 0.05.

To test the potential roles of these pathways in regulating PD-L1 expression upon pamiparib treatment, SW1990 cells were pre-treated with pamiparib for 12 h, followed by the treatment of specific inhibitors targeting these pathways, including the JAK-STAT signaling pathway (AG490) (**Figure 4A**), NF-kB signaling pathway (HY-N0274) (**Supplementary Figure 2A**), PI3K-AKT signaling pathway (LY294002) (**Supplementary Figure 2B**) and MAPK signaling pathway (SCH772984) (**Supplementary Figure 2C**). Results suggested an important role of JAK-STAT signaling pathway in mediating pamiparib-induced upregulation of PD-L1 (**Figure 4A**). Similar observations were noticed with inhibitors targeting PI3K-AKT or MAPK signaling pathway, but to a less extend (**Supplementary Figures 2B, C**). To further investigate the role of JAK-STAT signaling pathway, a specific inhibitor (stattic) against STAT3 was used and this treatment completely abolished the up-regulation of PD-L1 induced by pamiparib (**Figure 4B**).

We next investigated if pamiparib treatment could alter the activity of JAK-STAT signaling pathway. In SW1990 cells, pamiparib treatment activated JAK-STAT signaling pathway in a time-dependent manner, demonstrated by an increased level of phosphorylation in both JAK2 (**Figure 4C**) and STAT3 (**Figure 4D**). In addition, we also further explored the activation of PI3K-AKT signaling pathway and MAPK signaling pathway over time, and we found that the changes in phosphorylation of these two signaling pathways (**Supplementary Figures 2D, E**) did not coincide with the changes in PD-L1. Similar results were obtained *in vivo* by IHC staining of phospho-STAT3 (p-STAT3) (**Figure 4E**), with an increase upon pamiparib treatment (*P* < 0.05; **Figure 4F**). Together, the results demonstrated that pamiparib treatment induces PD-L1 expression *via* JAK2/STAT3 pathway, at least partially.

### Co-Administration of Pamiparib With PD-L1 Blocker Significantly Improves the Therapeutic Efficacy *In Vivo*

**Figure 5:**
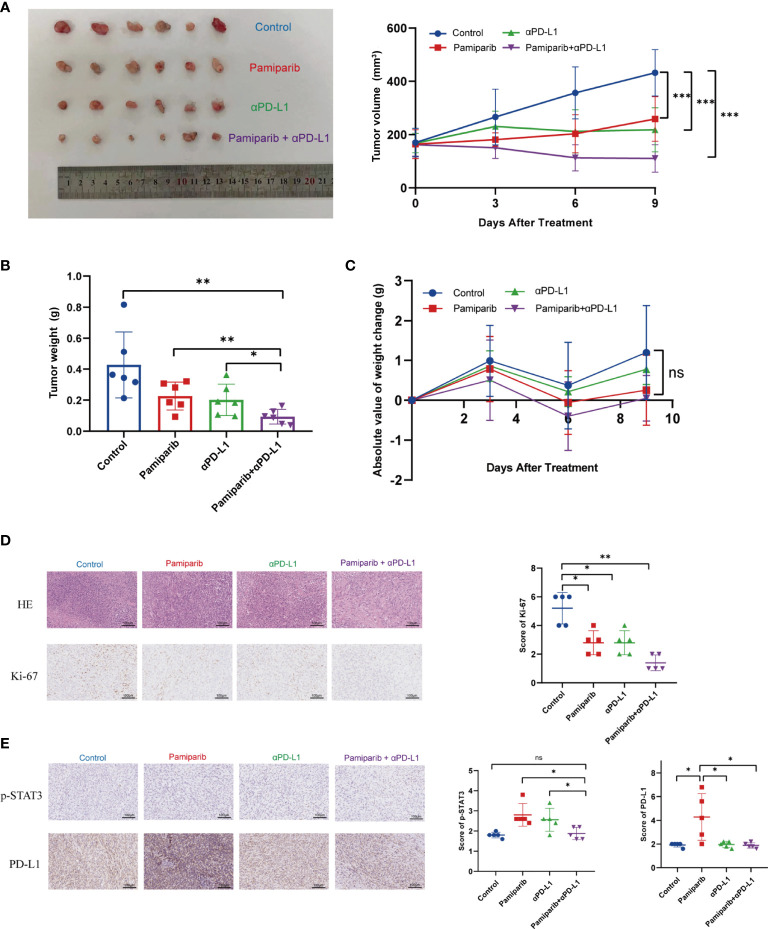
Co-administration of pamiparib with PD-L1 blocker significantly improves the therapeutic effect *in vivo*. **(A)** Tumor volume curves of C57 mice carrying Pan-02 allograft tumors in different treatment groups (n = 6) and tumor pictures at the end of treatment. **(B)** Tumor weight histograms of Pan-02 allograft mice in different treatment groups (n = 6). **(C)** Bodyweight curves of mice in four different treatment groups at the indicated time points after receiving treatment. Data analysis was performed by unpaired t-test. **(D)** Representative images of H&E and Ki-67 IHC staining of Pan-02 allograft tumors in mice from different treatment groups (n = 5). Scale bar, 100 μm. **(E)** IHC staining and scores showing consistent trends in PD-L1 and p-STAT3 changes were observed between the four groups of C57 (n = 5). Scale bar, 100 μm. *P < 0.05, **P < 0.01, ***P < 0.001, ns differences were not statistically significant.

Given the above observations that pamiparib treatment induces PD-L1 expressions, we next investigated whether blocking PD-L1 could enhance the anti-cancer effects of pamiparib in pancreatic cancer. A C57 mouse allograft tumor model using mouse pancreatic cancer cell line Pan-02 was utilized to assess their efficacy. When the tumor volume reached 100 mm^3^, mice were randomly divided into 4 groups for treatment (i.e., pamiparib monotherapy group, PD-L1 blocker monotherapy group, pamiparib and PD-L1 blocker combination group, and DMSO as a control group). Both pamiparib and anti-PD-L1 monotherapy significantly inhibited tumor growth. Interestingly, the combination therapy group achieved a better therapeutic efficacy compared to the monotherapy group (**Figures 5A, B**). The difference in bodyweight change was not statistically significant in mice receiving the combination treatment compared with mice in other experimental groups (**Figure 5C**). IHC staining of tumor specimens from mice showed that the combination treatment group had significantly fewer Ki-67 positive tumor cells than other groups (**Figure 5D**). These results indicated that the combination of PD-L1 blocker with pamiparib significantly inhibited the proliferative ability of tumors *in vivo*, i.e., enhanced the anti-cancer effect of pamiparib. In addition, the IHC staining of mice tumors in the pamiparib monotherapy group presented higher expression of PD-L1 and p-STAT3 than in the control group, and the expression of p-STAT3 and PD-L1 was reversed after combining pamiparib with anti-PD-L1 (**Figure 5E**). This phenomenon was consistent with the results in **Figure 4**.

### Combination Therapy With Pamiparib and PD-L1 Blocker Increases T-Cell Infiltrations

**Figure 6:**
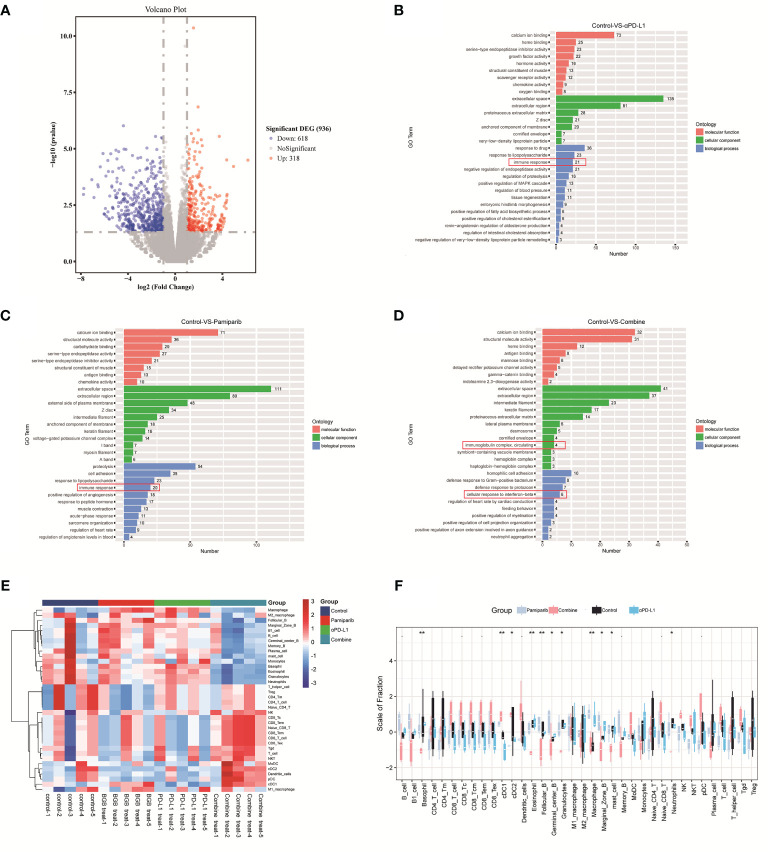
Bioinformatic analysis suggests that combination therapy with pamiparib and PD-L1 blocker alters the immune microenvironment. **(A)** Volcano plot for differential genes between control and combination treatment groups. **(B)** Bar plot showing the top 10 enriched CC, MF and BP terms between the PD-L1 inhibitor group (PD-L1) and the control group. **(C)** Bar plot showing the top 10 enriched CC, MF and BP terms between pamiparib monotherapy group (BGB290) and the control group. **(D)** Bar plot showing the top 10 enriched CC, MF and BP terms between PD-L1 inhibitor and pamiparib combination group (Combine) and the control group. Green indicates CC term, red indicates MF term and blue indicates BP term. The numbers indicate the numbers of enriched genes in each term. **(E)** Heat map of 22 immune cells expression in four different groups. **(F)** Boxplot of 22 immune cell infiltrations in four different groups. Data analysis was performed by unpaired t-test. *P < 0.05, **P < 0.01.

To understand the observations above, unbiased RNA-seq was performed to quantify the changes in gene expressions induced by pamiparib and/or anti-PD-L1 treatment. In total, there were 936 differentially expressed genes (DEGs) (defined as *P* < 0.05 and fold change ≥ 2) between the untreated controls and combination therapy groups (**Figure 6A**). Functional classification of DEGs was performed based on gene ontology (GO). The top 10 most significantly enriched cellular components (CC), molecular functions (MF), biological processes (BP) between control and PD-L1 blocker-alone groups, control and pamiparib-alone groups, and control and combination groups are presented in **Figures 6B–D** and **Supplementary Tables 2–4**. Interestingly, all 3 treatments significantly altered “immune-related” genes, suggesting that both monotherapy and combination therapy modulate genes related to the immune response. A significant number of DEGs in combination therapy were also enriched in the categories of “inflammatory response”, “innate immune response”, “neutrophil accumulation” and “response to IFN-β response” categories, suggesting that combination therapy significantly altered the expression of genes related to inflammation and the immune system.

We, therefore, used CIBERSORT analysis to calculate the abundance and immune score of 22 immune cell types. Both heat and box plots visualize that all components of CD8^+^ T cells were significantly higher in the combination treatment group compared to either the single-agent group or the control group (e.g., initial CD8^+^ T cells, memory CD8^+^ T cells, killer CD8^+^ T cells) (**Figures 6E, F** and **Supplementary Figure 3**). And the ratio of antigen-presenting cells and macrophages also had a significant upregulation.

**Figure 7:**
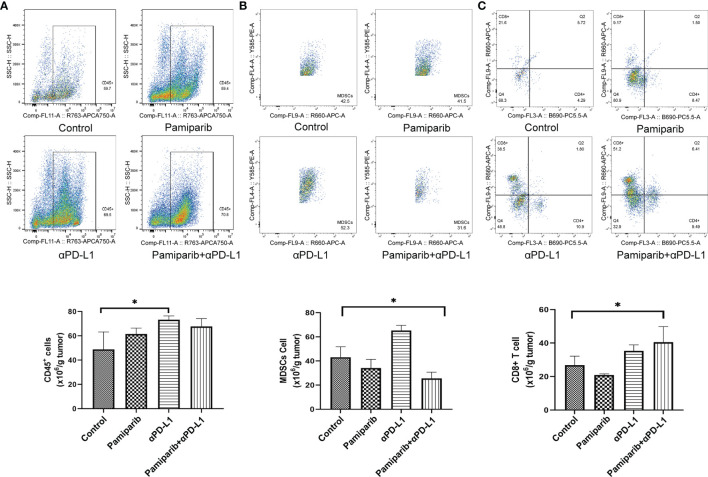
Flow cytometry analysis confirms that combination therapy with pamiparib and PD-L1 blocker alters the immune microenvironment. **(A–C)** Flow cytometry analysis to calculate the ratio values of CD45^+^ cells **(A)**, MDSC cells **(B)** and CD8^+^ T cells **(C)** per 1.0 g of tumor tissue. *P < 0.05.

To verify the above findings, flow cytometry was used to examine whether the combination treatment altered the tumor immune microenvironment. Increased infiltration of CD45^+^ immune cells in the combination group was observed by flow cytometry assay (**Figure 7A**, *P* < 0.05) and a significant decrease in myeloid-derived suppressor cells (MDSCs) infiltration (**Figure 7B**, *P* < 0.05). Similarly, the density of CD8^+^ T cells was increased in the combination treatment group (**Figure 7C**, *P* < 0.05).

Thus, the combination treatment of pamiparib and PD-L1 blocker significantly alters the tumor immune microenvironment, resulting in a significant upregulation of the proportion of CD8^+^ T cells.

## Discussion

The prognosis of pancreatic cancer remains poor and no significant improvement has been achieved in the last 2 decades. Innovative findings are urgently needed to improve the 5-year survival rate of pancreatic cancer patients. Many studies have shown that the unique biological behavior of pancreatic cancer is related to the tumor microenvironment (29–31). The immunosuppressive microenvironment of pancreatic cancer is highly heterogeneous, posing a challenge for immunotherapy. Immunotherapies that have received FDA approval for use in other tumors to date have little to no efficacy against this cancer. The problem lies in its strikingly immunosuppressive and “immune privileged” tumor microenvironment, where few patients exhibit robust T-cell infiltration (32). Thus, pancreatic cancer has been classically described as a “cold” tumor because it is characterized by a relative paucity of intratumoral CD8^+^ T cells (33). A shift in the immunosuppressive microenvironment of the tumor contributes to the response to tumor immunotherapy (34). Future treatments for pancreatic cancer will likely be based on the development of new therapies based on the genomic and proteomic identification of cellular/immune processes and molecular pathways as therapeutic targets (35).

**Figure 8:**
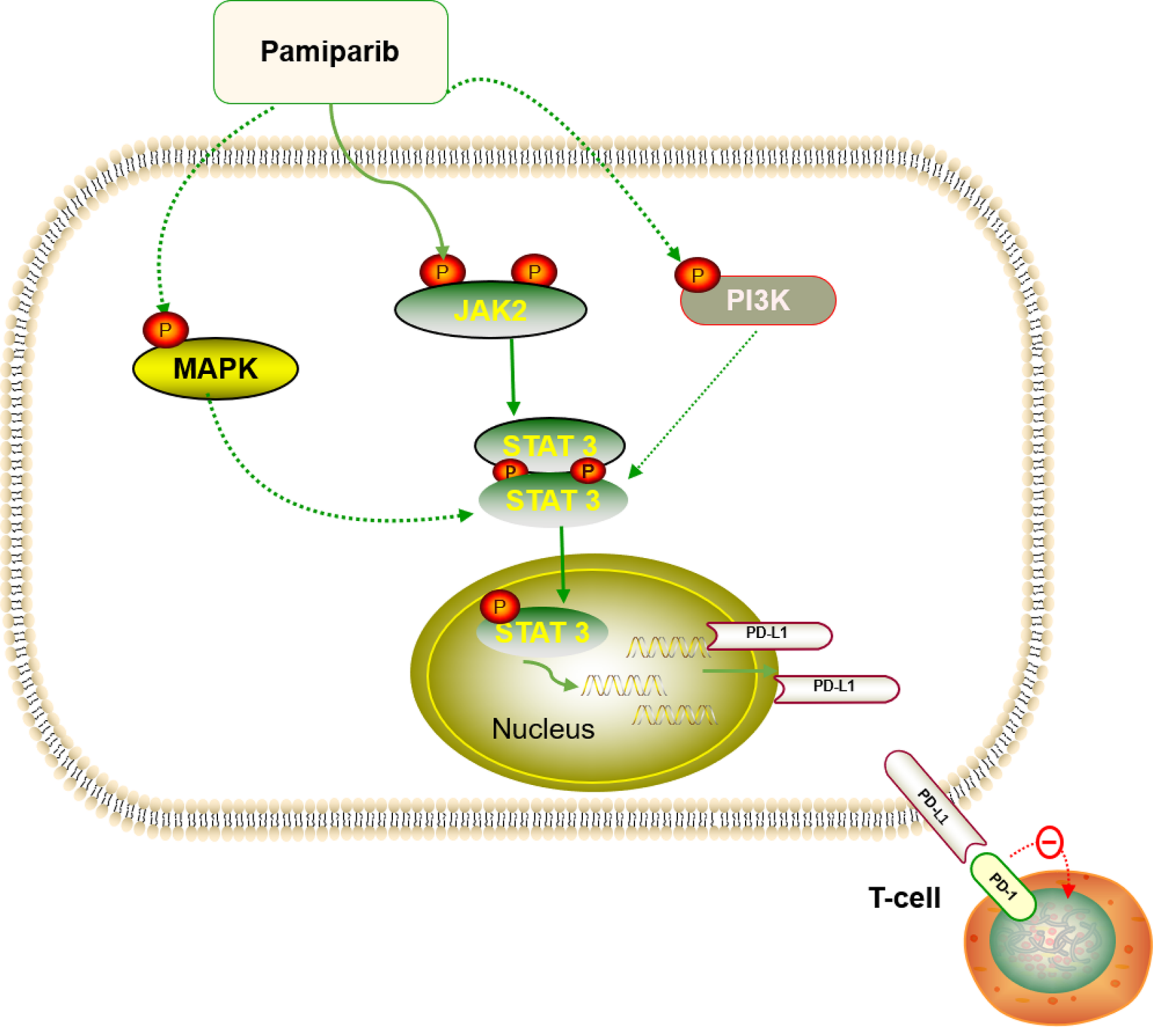
Diagram summarizing that pamiparib treatment induces PD-L1 expression mainly via JAK2/STAT3 in pancreatic cancer (details provided in the *Discussion* section).

Together with other reports in breast cancer (36), ovarian cancer (37), and non-small cell lung cancer (38), our study shows that simultaneous inhibition of PARP and PD-L1 confers therapeutic benefits. However, although the cytotoxic effects of PARPis have been well studied, the role of PARPis regarding how they modulate cancer-related immunity in pancreatic cancer remains largely unknown. Previously, it was revealed that PARPis upregulate PD-L1 expression through different pathways in breast and ovarian cancer, making the combination of the two more effective (36, 39). In this study, we demonstrate that pamiparib upregulates PD-L1 expression through the JAK2/STAT3 pathway, at least partially (**Figure 8**), and that PD-L1 blockers enhance the effects of pamiparib *in vitro* and *in vivo*. Interestingly, although it has been suggested that tumors with *BRCA* mutations are sensitive to PARPis (40, 41), findings from our study indicate that PD-L1 induction PARPis by is not dependent on *BRCA* status, since similar results were obtained from the human pancreatic cancer cell line SW1990 and mouse pancreatic cancer cell line Pan-02, which have a *BRCA1* mutation, as well as the human pancreatic cancer cell line BxPc-3 does not have mutations in either *BRCA1* or *BRCA2* (https://cancer.sanger.ac.uk/cell_lines/).

We show that combination treatment of PAPRi and anti-PD-L1 induces tumor regression in immunocompetent mice. The combination treatment increases the infiltration of CD8^+^ cytotoxic T cells and decreases the infiltration of MDSCs. As a result, the relatively poor presence of CD8^+^ T cells in the immune microenvironment of pancreatic cancer is improved. Practically, these combinations are well tolerated in patients with combinations of 3 different PARPi [Olaparib (42), niraparib (43) and BGB-290 (44)] and PD-L1 or PD-1 antibodies being tested in a variety of cancer types (NCT02657889, NCT02484404 and NCT02660034). In this study, there are some limitations, including using a subcutaneous tumor model and no PD-L2 expression investigated. It has been suggested that orthotopic tumor models are more clinically relevant than their subcutaneous counterparts, although the latter is also widely used to study the immune microenvironment of pancreatic cancer (45, 46). Additionally, we reported that PD-L1 blockers enhance the effects of pamiparib *in vitro* and *in vivo*. The potential effects on PD-L2 are interesting and merit further investigation. Despite these limitations, our findings suggest a realistic scenario of a prospective clinical trial in pancreatic cancer patients with the combination therapy of PARP inhibitor and anti-PD-L1/PD-1.

## Materials and Methods

### Mice, Cell Lines, and Reagents

All procedures were approved by the Institutional Animal Care and Use Committee of Tongji Medical College, Huazhong University of Science and Technology (approval number: TJH-201908003). Female C57BL/6 mice and BALB/c nude mice (6 weeks old) were obtained from the Jiangsu Jicui Pharmachem Experimental Animal Center. And the mice were housed in 5 animals per cage under standard laboratory conditions and fed with sterilized food and water. Mouse Pan02 and human-derived SW1990 and BxPC-3 pancreatic cancer cell lines were obtained from the Oncology Laboratory of Wuhan Tongji Hospital. Primary T cells were obtained from Wuhan Bio-Raid Biotechnology Co. Cancer cells were cultured in Dulbecco’s modified Eagle’s medium (DMEM) (HyClone, Logan, UT) supplemented with 10% fetal bovine serum (FBS) (Gibco, 10270-106, GER). Primary T cells were cultured by Metanni’s Tex medium while activated with 100ng/mL CD3 antibody, 100ng/mL CD28 antibody and 10ng/mL IL2 (#317303; #302913; #589102, BioLegend). All cells were cultured in a humidified incubator at 37°C and 5% CO2. Pamiparib is one of PARPis, which was presented by BeiGene. No mycoplasma contamination was detected in the cell lines used. Anti-PD-L1 antibody (clone 10 F.9G2, Cat#BE0101) was purchased from BioXcell (West Lebanon, NH, USA).

### Western Blotting

The primary antibodies are PD-L1 (CST #13684, Cell Signaling Technology), PD-L1 (17952- 1-AP, Santa Cruz), STAT3 (CST #9139, Cell Signaling Technology), phosphorylated STAT3 (CST #9145, Cell Signaling Technology), JAK2 (17670-1-AP, Proteintech), phosphorylated JAK2 (CST #4406, Cell Signaling Technology), AKT (10176-2-AP, Proteintech), phosphorylated AKT (66444-1-Ig, Proteintech), ERK (CST #4696, Cell Signaling Technology), phosphorylated ERK (CST #3510, Cell Signaling Technology), PARP-1 (sc-8007, Santa Cruz), and GAPDH (60004-1-Ig, Proteintech). Goat anti-rabbit antibody and mouse anti-rabbit antibody conjugated to HRP were purchased from Biosharp. all antibodies and reagents were stored and used according to the manufacturer’s instructions. Briefly, tissues were homogenized, mixed with 5X loading buffer and boiled until denaturation. Proteins were separated by sodium dodecyl sulfate-polyacrylamide gel electrophoresis and transferred to polyvinylidene difluoride membranes. Membranes are sealed with 5% skim milk and incubated overnight at 4°C with the primary antibody. The membranes were then washed, incubated with secondary antibodies for 1 hour at room temperature, and visualized with SuperSignal West Pico plus chemiluminescent substrate (Thermo Fisher Scientific, Waltham, MA).

### Nude Mice Experiment

A dose of the drug was weighed and dissolved in a mixture of 10% DMSO, 5% Tween-80, 85% saline to form a solution of 12 mg/ml, which could be used for subsequent animal experiments in which mice were fed. SW1990 cell line was resuscitated, the cell status was adjusted to logarithmic cell growth, it was digested, centrifuged and the cell number was adjusted to 2x10^7^/ml using serum-free medium. week-old male BALB/c nude mice were taken and 100ul of SW1990 cell suspension was injected into the right lower back of each nude mouse (i.e., the number of injections was 2 × 10^6^). Tumor formation was observed, and when the tumor volume of mice was greater than 100 mm^3^ [Tumor volume = (length × width × width)/2], the tumor size was sorted in order from largest to smallest, and the mice were sequentially divided into the experimental and control groups of pamiparib group. Mice in the experimental group were given oral pamiparib (3 mg/kg) twice daily, while mice in the control group were not given any special treatment. After 3 weeks of administration, the mice were sacrificed and the tumor tissues were removed. The tissues were labeled and fixed in 4% paraformaldehyde. Paraffin-embedded tissues were used for subsequent experiments.

### Drug Treatments in Mice

The cells were centrifuged after digestion and resuspended in the appropriate serum-free medium, and the density was adjusted to 2 × 10^7^/ml after measuring the cell density with an automatic counting plate. All mice were housed in an SPF class mouse rearing room and fed with water freely. All animals were housed and operated following the relevant regulatory and ethical requirements for experimental animals. When the tumor volume was larger than 100 mm^3^, the mice were sorted according to the size of the tumor from the largest to the smallest, and after ear tagging, each mouse was randomly assigned to each experimental group and pair according to the principle of random assignment, and they were randomly divided into 4 groups (6 mice in each group). The mice were divided into 4 groups: (i) control group; (ii) PD-L1 inhibitor group; (iii) pamiparib monotherapy group; and (iv) PD-L1 inhibitor and pamiparib combination group. The mice in the pamiparib group were fed twice daily with 3 mg/kg each time. pamiparib mice in the PD-L1 group were injected intraperitoneally with murine PD-L1 antibody at 10 mg/kg each time every 3 d. Both pamiparib and PD-L1 were administered in the same way as in the first two groups. Tumor volume and body weight were measured by digital calipers and electronic scales every 3 days. After 4 injections of anti-mouse PD-L1 antibody, the mice were sacrificed, tumor tissues were removed and photographed. A straightedge needs to be placed at the time of photographing, which can be used to calculate the tumor volume later. The tissues were processed differently, and one part was collected for flow cytometry detection. One part was fixed in 4% paraformaldehyde and embedded in paraffin. Paraffin-embedded tissue sections are stained for H&E and IHC. A portion was frozen in liquid nitrogen for RNA-seq.

### Transfections

After digesting the SW1990 cell line in a logarithmic growth phase, adjust the cell number to 1×10^5^/ml using the medium in the absence of resistance, inoculate 1 ml of cell suspension in each well of the 6-well plate, and wait for the cell fusion to be 30-50% for subsequent transfection experiments. (Note: When spreading the plate, the cells should be digested and mixed completely to avoid cell pile-up growth. Dilute si*PARP1* (final concentration of transfected cells is 50nM) with 50 ul Opti-MEM and mix by gently blowing 3~5 times. Mix the transfection reagent and si*PARP1* dilution by gently inverting, dilute 1.0 ul LipofectamineTM 2000 with 50 ul Opti-MEM, gently blow 3~5 times to mix, and let stand at room temperature for 5 min. The transfection complex was added to 6-well cell plates, 100μL/well, and the plates were gently shaken before and after to mix well. The plates were incubated at 37°C, 5% CO2, in an incubator for 18~48 h. After transfection for 4~6 h, the media could be changed to fresh ones.

### Flow Cytometry

Tumor sections were weighed, cut into small pieces, and digested with Mouse Tumor Dissociation Kit (Cat# 130-096-730, Miltenyi Biotec) enzyme cocktail solution at 37°C for 30 minutes. Add PBS containing 1% fetal bovine serum to stop the reaction. Cells were pelleted at 1200 rpm for 5 min at 4°C, resuspended in phosphate-buffered saline, and mashed through a 70 μm cell filter. To detect the lymphocyte component of the infiltrating tumor microenvironment, cells were stained with the antibodies listed in **Supplementary Table 5** according to the protocol for flow cytometry. Data were collected with a CytoFLEX S (Beckman Coulter) or BD LSRII cytometer and analyzed with FlowJo software (version 7.6; Tree Star, Ashland, OR, USA). Cell populations were quantified by gating from single-stained positive controls and fluorescent minus one (FMO) controls.

### RNA Sequencing

RNA sequencing was performed by Illumina HiSe, and total RNA from each sample was extracted using TRIzol Reagent (Invitrogen)/RNeasy Mini Kit (Qiagen)/other kits for the preparation of the following libraries. The PCR products were washed with beads, validated with Qsep100 (Bioptic, Taiwan, China), and quantified with a Qubit 3.0 fluorometer (Invitrogen, Carlsbad, CA, USA). Libraries of different indices were multiplexed and loaded on an Illumina HiSeq instrument (Illumina, San Diego, CA, USA) according to the manufacturer’s instructions. Sequencing was performed using a 2x150bp paired-end (PE) configuration; image analysis and base calling were performed on the HiSeq instrument by HiSeq Control Software (HCS) + OLB + GAPipeline-1.6 (Illumina). Sequences were processed and analyzed by GENEWIZ. The CIBERSORT analysis used for the subsequent calculation of the abundance and immune score of the 22 immune cells was drawn by ggplot. RNA-seq data have been deposited in NCBI database (accession code PRJNA724048).

### Immunohistochemistry

After antigen repair, samples were placed in 5% BSA and incubated for 20 min for closure. At 4°C with the corresponding primary antibodies [PD-L1 (1:200, CST #13684, Cell Signaling Technology), phospho-STAT3 (1:200, CST #9145, Cell Signaling Technology), Ki-67 (1:200, ab16667, Abcam)] were incubated overnight. After overnight incubation, sections were rinsed 3 times with PBS solution for 5 min each. Sections were placed in antibody solution, incubated with secondary antibody at 37°C for 30 min, and then rinsed 3 times with PBS solution for 5 min each. Stained, and blocking, IHC results were scored immunohistochemically by 2 independent observers.

### Bioinformatic Analysis

We searched for Pancreatic cancer in c-Bioportal, then searched for genes positively correlated with CD274 co-expression, then imported the genes with cor ≥ 0.4 into STRING, constructed the co-expression network and then analyzed them. We obtained pathways associated with PD-L1 regulation from KEGG analysis.

### Statistical Analysis

Statistical analyses were conducted using SPSS version 26.0 software (SPSS Inc., Chicago, USA) and GraphPad Prism software (GraphPad version 8.0). The IHC results were analyzed by Pearson χ^2^ test. Data were displayed as mean ± SEM. Differences between variables were analyzed by one-way ANOVA or two-tailed Student’s *t*-test for *P*-values. Differences were considered statistically significant at *P* < 0.05. Each experiment was repeated at least 3 times.

## Data Availability Statement

The original contributions presented in the study are publicly available. This data can be found here: https://www.ncbi.nlm.nih.gov/bioproject/PRJNA724048. Further inquiries can be directed to the corresponding authors.

## Ethics Statement

The animal study was reviewed and approved by the Institutional Animal Care and Use Committee of Tongji Medical College, Huazhong University of Science and Technology.

## Author Contributions

YMZ, HX and YHW conceived, designed, and managed the study; YLW, KZ, HX, YH, XC, YLZ, WQ, JS, and RC performed the experiments; YLW, KZ, HX, HQ, XY, YHW and YMZ drafted the manuscript; All authors approved the final manuscript.

## Funding

This project was supported by the National Natural Science Foundation of China [81772827] for YHW; the Natural Science Foundation of Hubei Province [2021CFB372] for HX.

## Conflict of Interest

The authors declare that the research was conducted in the absence of any commercial or financial relationships that could be construed as a potential conflict of interest.

## Publisher’s Note

All claims expressed in this article are solely those of the authors and do not necessarily represent those of their affiliated organizations, or those of the publisher, the editors and the reviewers. Any product that may be evaluated in this article, or claim that may be made by its manufacturer, is not guaranteed or endorsed by the publisher.
