# Supplementary material for: PARP Inhibitor Upregulates PD-L1 Expression and Provides a New Combination Therapy in Pancreatic Cancer
Source: Front Immunol. 2021 Dec 17;12:762989. doi: 10.3389/fimmu.2021.762989 (PMC8718453; doi:10.3389/fimmu.2021.762989)
Supplement: Supplementary file 1 [file DataSheet_1.pdf]

PARP Inhibitor Upregulates PD-L1 Expression and Provides a New Combination Therapy In Pancreatic Cancer

Supplementary Materials

Supplementary Figures

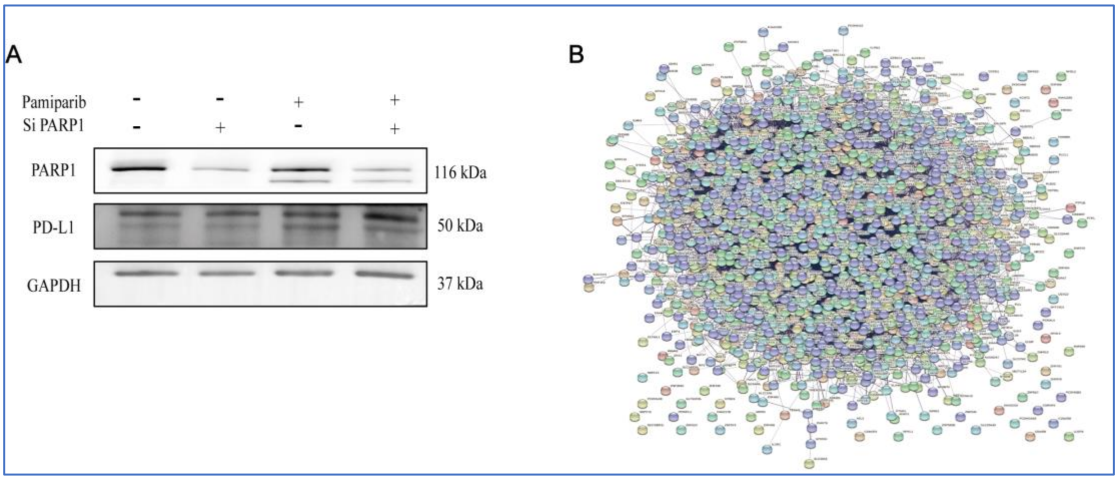

Supplementary Figure 1. Effects of PARPi treatment on PD-L1 expression. (A) PARP1 mRNA levels do not affect the upregulation of PD-L1 induced by PARPi. (B) PPI co-expression network analysis in genes associated with PD-L1 upregulation in pancreatic cancer cells.

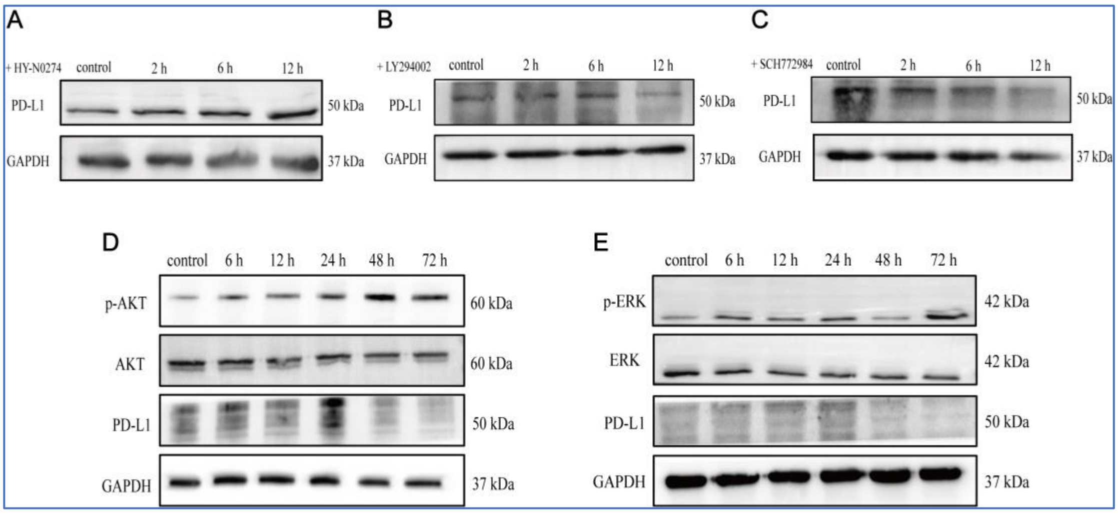

Supplementary Figure 2. PARP inhibitor treatment induces PD-L1 expression via JAK2/STAT3 pathway. (A-C) Cells were pretreated with pamiparib (100 μM) for 12 hrs and PD-L1 expression was assessed by western blotting after treatment with 20 μM of HY-N0274 (A), LY294002 (B) or SCH772984 (C), respectively, for 24 hrs. (D and E) Expression of AKT, p-AKT, ERK, and p-ERK were detected by western blotting in SW1990 cells following treatment with pamiparib (100 μM) for the indicated times. GAPDH was used as a loading control.

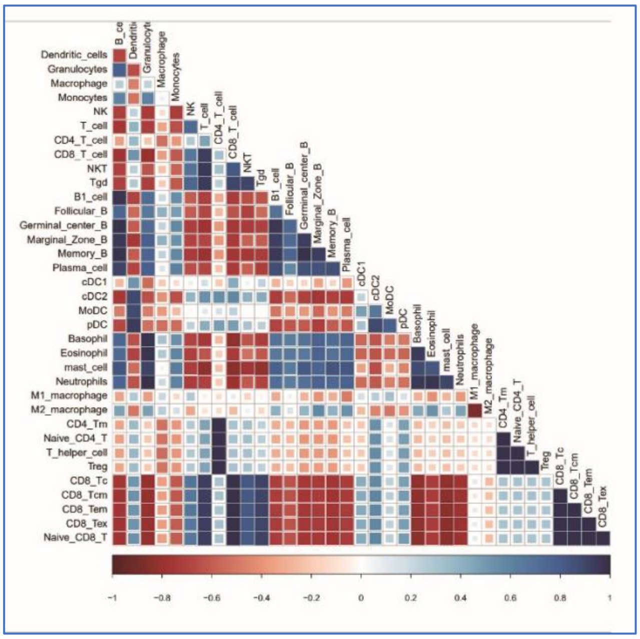

Supplementary Figure 3. Bioinformatic analysis suggests that combination therapy with PARPi and PD-L1 blocker alters the immune microenvironment.

Supplementary Tables

Supplementary Table 1. KEGG enrichment pathways of genes in PPI co-expression network.

#term ID	term description	observed gene count	background gene count	strength	false discovery rate
hsa04014	Ras signaling pathway	42	228	0.38	6.03E-05
hsa04151	PI3K-Akt signaling pathway	54	348	0.31	0.00014
hsa05200	Pathways in cancer	72	515	0.26	0.00014
hsa04659	Th17 cell differentiation	24	102	0.49	0.00017
hsa04658	Th1 and Th2 cell differentiation	20	88	0.47	0.00094
hsa04630	Jak-STAT signaling pathway	29	160	0.38	0.00094
hsa04660	T cell receptor signaling pathway	21	99	0.44	0.0013
hsa04010	MAPK signaling pathway	43	293	0.28	0.0014
hsa04012	ErbB signaling pathway	17	83	0.43	0.0043
hsa04668	TNF signaling pathway	20	108	0.38	0.0043
hsa04140	Autophagy - animal	22	125	0.36	0.0043
hsa04620	Toll-like receptor signaling pathway	19	102	0.39	0.0051
hsa05212	Pancreatic cancer	15	74	0.42	0.0074
hsa04650	Natural killer cell mediated cytotoxicity	20	124	0.32	0.0138
hsa04064	NF-kappa B signaling pathway	16	93	0.35	0.0186
hsa04662	B cell receptor signaling pathway	13	71	0.38	0.0237
hsa04210	Apoptosis	20	135	0.29	0.0252
hsa04144	Endocytosis	30	242	0.21	0.0378
hsa04066	HIF-1 signaling pathway	15	98	0.3	0.0456

Supplementary Table 2. The top 10 most significantly enriched CC, MF, BP between control and PD-L1 blocker-alone groups.

category	term	ontology	numDEInCat	numInCat	over_represented_pvalue	over_represented_FDR	GeneNumber(Up)	GeneNumber(Down)
GO:0005615	extracellular space	CC	135	1054	9.92E-41	1.34E-36	112	23
GO:0005576	extracellular region	CC	81	932	7.77E-14	5.26E-10	75	6
GO:0005578	proteinaceous extracellular matrix	CC	28	171	3.74E-09	8.44E-06	26	2
GO:0031225	anchored component of membrane	CC	20	112	2.02E-08	2.74E-05	19	1
GO:0030018	Z disc	CC	21	114	6.49E-08	7.32E-05	19	2
GO:0034361	very-low-density lipoprotein particle	CC	7	19	2.29E-06	0.001408	7	0
GO:0001533	cornified envelope	CC	7	23	5.31E-06	0.002768	6	1
GO:0042627	chylomicron	CC	5	11	1.68E-05	0.006503	5	0
GO:0034366	spherical high-density lipoprotein particle	CC	4	8	3.59E-05	0.012154	4	0

GO:0072562	blood microparticle	CC	3	3	8.57E-05	0.022236	3	0
GO:0005509	calcium ion binding	MF	73	758	5.8E-12	2.62E-08	64	9
GO:0004867	serine-type endopeptidase inhibitor activity	MF	23	146	3.43E-09	8.44E-06	22	1
GO:0008307	structural constituent of muscle	MF	13	35	5.12E-09	9.91E-06	11	2
GO:0005179	hormone activity	MF	16	107	8.91E-08	8.69E-05	13	3
GO:0020037	heme binding	MF	25	207	8.98E-08	8.69E-05	24	1
GO:0019825	oxygen binding	MF	8	23	3.07E-07	0.000277	8	0
GO:0008083	growth factor activity	MF	22	168	5.66E-07	0.000416	17	5
GO:0005044	scavenger receptor activity	MF	12	45	5.83E-07	0.000416	11	1
GO:0008009	chemokine activity	MF	9	46	9.06E-06	0.00438	6	3
GO:0070653	high-density lipoprotein particle	MF	3	3	1.03E-05	0.004502	3	0

	receptor binding							
GO:0010951	negative regulation of endopeptidase activity	BP	21	116	6.96E-10	2.36E-06	19	2
GO:0030162	regulation of proteolysis	BP	16	75	1.11E-08	1.87E-05	15	1
GO:0042246	tissue regeneration	BP	11	29	1.51E-08	2.28E-05	11	0
GO:0010873	positive regulation of cholesterol esterification	BP	6	8	6.03E-08	7.32E-05	6	0
GO:0006955	immune response	BP	21	155	3.35E-07	0.000283	14	7
GO:0032496	response to lipopolysaccharide	BP	23	174	4.86E-07	0.000387	16	7
GO:0030300	regulation of intestinal cholesterol absorption	BP	4	4	9.57E-07	0.000648	4	0
GO:0043410	positive regulation of	BP	13	62	1.65E-06	0.001065	11	2

	MAPK cascade							
GO:0002018	renin-angiotensin regulation of aldosterone production	BP	4	4	2.85E-06	0.001676	4	0
GO:0045723	positive regulation of fatty acid biosynthetic process	BP	6	12	3.85E-06	0.002174	6	0

Supplementary Table 3. The top 10 most significantly enriched CC, MF, BP between control and pamiparib-alone groups.

category	term	ontology	numDEInCat	numInCat	over_represented_pvalue	over_represented_FDR	GeneNumber (Up)	GeneNumber (Down)
GO:0005615	extracellular space	CC	111	1054	9.54E-23	1.29E-18	30	81
GO:0030018	Z disc	CC	34	114	2.18E-18	1.48E-14	3	31
GO:0005882	intermediate filament	CC	25	77	2.53E-17	1.14E-13	1	24
GO:0009897	external side of plasma membrane	CC	48	289	3.15E-16	8.54E-13	6	42
GO:0045095	keratin filament	CC	16	38	4.58E-13	1.03E-09	1	15
GO:0005576	extracellular region	CC	80	932	2.29E-11	3.88E-08	15	65
GO:0008076	voltage-gated potassium channel complex	CC	14	54	1.22E-07	0.000103	0	14
GO:0032982	myosin filament	CC	7	13	7.74E-07	0.000524	0	7

GO:0031225	anchored component of membrane	CC	18	112	1.49E-06	0.000875	0	18
GO:0031672	A band	CC	6	11	7.2E-06	0.003165	0	6
GO:0005198	structural molecule activity	MF	36	159	1.47E-16	4.97E-13	2	34
GO:0030246	carbohydrate binding	MF	29	161	1.28E-11	2.48E-08	1	28
GO:0008307	structural constituent of muscle	MF	15	35	3.92E-11	5.89E-08	1	14
GO:0005509	calcium ion binding	MF	71	758	5.24E-10	7.1E-07	10	61
GO:0003823	antigen binding	MF	13	56	8.66E-10	1.07E-06	0	13
GO:0004252	serine-type endopeptidase activity	MF	27	207	5.39E-08	4.87E-05	4	23
GO:0004867	serine-type endopeptidase inhibitor activity	MF	21	146	3.84E-07	0.000306	12	9

GO:0008009	chemokine activity	MF	10	46	2.98E-06	0.001554	0	10
GO:0031432	titin binding	MF	6	15	1.1E-05	0.004037	0	6
GO:0008201	heparin binding	MF	23	279	2.47E-05	0.007294	1	22
GO:0006936	muscle contraction	BP	13	38	2.51E-09	2.83E-06	0	13
GO:0043434	response to peptide hormone	BP	17	82	3.5E-08	3.65E-05	8	9
GO:0006508	proteolysis	BP	54	580	5.33E-08	4.87E-05	9	45
GO:0006953	acute-phase response	BP	11	38	4.61E-07	0.000347	6	5
GO:0045766	positive regulation of angiogenesi s	BP	18	105	7.01E-07	0.0005	0	18
GO:0002027	regulation of heart rate	BP	9	28	1E-06	0.000645	1	8
GO:0007155	cell adhesion	BP	35	308	1.16E-06	0.000712	4	31

GO:0045214	sarcomere organization	BP	10	31	1.66E-06	0.000937	1	9
GO:0032496	response to lipopolysaccharide	BP	23	174	1.84E-06	0.000996	3	20
GO:0006955	immune response	BP	20	155	5.05E-06	0.002533	2	18

Supplementary Table 4. The top 10 most significantly enriched CC, MF, BP between control and combination groups.

category	term	ontology	numDEInCat	numInCat	over_represented_pvalue	over_represented_FDR	GeneNumber(Up)	GeneNumber(Down)
GO:0005882	intermediate filament	CC	23	77	8.41E-22	1.03E-17	2	21
GO:0045095	keratin filament	CC	17	38	1.51E-19	6.8E-16	2	15
GO:0031838	haptoglobin-hemoglobin complex	CC	3	3	3.82E-06	0.007448	0	3
GO:0005615	extracellular space	CC	41	1054	9.62E-06	0.015914	11	30
GO:0005578	proteinaceous extracellular matrix	CC	14	171	2.44E-05	0.027392	4	10
GO:0005576	extracellular region	CC	37	932	2.47E-05	0.027392	12	25
GO:0042571	immunoglobulin complex, circulating	CC	4	16	5.66E-05	0.054739	0	4
GO:0030057	desmosome	CC	5	25	0.000167	0.138088	0	5
GO:0005833	hemoglobin complex	CC	3	9	0.000187	0.140881	0	3
GO:0020005	symbiont-containing	CC	3	6	0.000207	0.141056	3	0

	vacuole membrane							
GO:0005198	structural molecule activity	MF	31	159	1.52E-21	1.03E-17	3	28
GO:0003823	antigen binding	MF	8	56	1.57E-06	0.004242	0	8
GO:0005537	mannose binding	MF	6	22	3.85E-06	0.007448	0	6
GO:0005509	calcium ion binding	MF	32	758	8.71E-05	0.078618	9	23
GO:0045295	gamma-catenin binding	MF	4	18	0.000413	0.254147	0	4
GO:0033754	indoleamine 2,3-dioxygenase activity	MF	2	2	0.000445	0.262191	2	0
GO:0005251	delayed rectifier potassium channel activity	MF	5	32	0.000657	0.292056	0	5
GO:0020037	heme binding	MF	12	207	0.00067	0.292056	5	7
GO:0019825	oxygen binding	MF	4	23	0.000697	0.292056	1	3

GO:0043177	organic acid binding	MF	2	3	0.00084	0.325165	0	2
GO:0042832	defense response to protozoan	BP	7	23	3.22E-07	0.00109	6	1
GO:0050830	defense response to Gram-positive bacterium	BP	8	56	1.06E-05	0.015914	5	3
GO:0035458	cellular response to interferon-beta	BP	6	27	1.69E-05	0.022955	6	0
GO:0031643	positive regulation of myelination	BP	4	9	2.63E-05	0.027392	0	4
GO:0070488	neutrophil aggregation	BP	2	2	0.000173	0.138088	0	2
GO:0031346	positive regulation of cell projection organization	BP	3	7	0.000208	0.141056	2	1
GO:0007156	homophilic cell adhesion	BP	10	126	0.000469	0.263166	2	8

GO:0048842	positive regulation of axon extension involved in axon guidance	BP	2	2	0.000503	0.263166	1	1
GO:0007631	feeding behavior	BP	4	19	0.000512	0.263166	1	3
GO:0086091	regulation of heart rate by cardiac conduction	BP	4	18	0.000525	0.263166	0	4

Supplementary Table 5. List of antibodies used for flow cytometry.

Antibody	Source	Catalog
BD Pharmingen APC-CY7 Rat Anti-Mouse CD45	BD Pharmingen	557659
BD Pharmingen PE Hamster Anti-Mouse CD3e	BD Pharmingen	553063
BD Horizon BB700 Rat Anti-Mouse CD4	BD Pharmingen	566408
BD Pharmingen APC Rat Anti-Mouse CD8a	BD Pharmingen	561093
BD Pharmingen APC Rat Anti-CD11b	BD Pharmingen	561690
BD Pharmingen PE Hamster Anti-Mouse Gr-1	BD Pharmingen	553128
BioLegend PE anti-human CD274	BioLegend	329705
BioLegend PE anti-mouse CD274	BioLegend	124307
